# Design and validation of a high-density single nucleotide polymorphism array for the Eastern oyster (*Crassostrea virginica*)

**DOI:** 10.1093/g3journal/jkad071

**Published:** 2023-03-26

**Authors:** Amanda Xuereb, Rodrigo Marín Nahuelpi, Eric Normandeau, Charles Babin, Martin Laporte, André Mallet, José M Yáñez, Martin Mallet, Louis Bernatchez

**Affiliations:** Département de Biologie, Institut de Biologie Intégrative et des Systèmes (IBIS), Université Laval, 1030 Av. de la Médecine, Québec, QC, G1V0A6, Canada; Facultad de Ciencias Veterinarias y Pecuarias, Universidad de Chile, Santa Rosa 11735, La Pintana, Santiago, 8820808, Chile; Doctorado en Acuicultura, Programa Cooperativo Universidad de Chile, Universidad Católica del Norte, Pontificia Universidad Católica de Valparaíso, Instituto de Nutrición y Tecnología de los Alimentos, El Líbano 5524, Macul, Santiago 7830417, Chile; Département de Biologie, Institut de Biologie Intégrative et des Systèmes (IBIS), Université Laval, 1030 Av. de la Médecine, Québec, QC, G1V0A6, Canada; Département de Biologie, Institut de Biologie Intégrative et des Systèmes (IBIS), Université Laval, 1030 Av. de la Médecine, Québec, QC, G1V0A6, Canada; Département de Biologie, Institut de Biologie Intégrative et des Systèmes (IBIS), Université Laval, 1030 Av. de la Médecine, Québec, QC, G1V0A6, Canada; Ministère de l'Environnement, de la Lutte contre les changements climatiques, de la Faune et des Parcs, 880 Ch Ste-Foy, Québec, QC G1S 4X4, Canada; L’Étang Ruisseau Bar Ltd., 111 Rue Pointe-Brûlée, Shippagan, NB, E8S 3H9, Canada; Facultad de Ciencias Veterinarias y Pecuarias, Universidad de Chile, Santa Rosa 11735, La Pintana, Santiago, 8820808, Chile; L’Étang Ruisseau Bar Ltd., 111 Rue Pointe-Brûlée, Shippagan, NB, E8S 3H9, Canada; Département de Biologie, Institut de Biologie Intégrative et des Systèmes (IBIS), Université Laval, 1030 Av. de la Médecine, Québec, QC, G1V0A6, Canada

**Keywords:** *Crassostrea virginica*, Eastern oyster, aquaculture, SNP array

## Abstract

Dense single nucleotide polymorphism (SNP) arrays are essential tools for rapid high-throughput genotyping for many genetic analyses, including genomic selection and high-resolution population genomic assessments. We present a high-density (200 K) SNP array developed for the Eastern oyster (*Crassostrea virginica*), which is a species of significant aquaculture production and restoration efforts throughout its native range. SNP discovery was performed using low-coverage whole-genome sequencing of 435 F1 oysters from families from 11 founder populations in New Brunswick, Canada. An Affymetrix Axiom Custom array was created with 219,447 SNPs meeting stringent selection criteria and validated by genotyping more than 4,000 oysters across 2 generations. In total, 144,570 SNPs had a call rate >90%, most of which (96%) were polymorphic and were distributed across the Eastern oyster reference genome, with similar levels of genetic diversity observed in both generations. Linkage disequilibrium was low (maximum *r*^2^ ∼0.32) and decayed moderately with increasing distance between SNP pairs. Taking advantage of our intergenerational data set, we quantified Mendelian inheritance errors to validate SNP selection. Although most of SNPs exhibited low Mendelian inheritance error rates overall, with 72% of called SNPs having an error rate of <1%, many loci had elevated Mendelian inheritance error rates, potentially indicating the presence of null alleles. This SNP panel provides a necessary tool to enable routine application of genomic approaches, including genomic selection, in *C. virginica* selective breeding programs. As demand for production increases, this resource will be essential for accelerating production and sustaining the Canadian oyster aquaculture industry.

## Introduction

Aquaculture production is growing faster than any other food production sector globally. It recently surpassed capture fisheries landings for all harvested species groups (FAO, 2020) and will become increasingly important as food demands increase (Garlock *et al.* 2020). Aquaculture production with a low environmental cost will be especially important, and shellfish are an especially attractive group due to their low carbon footprint (Jones *et al.* 2022; Ray *et al.* 2019), minimal inputs (i.e. supplementary feeding), and ecological benefits such as nutrient cycling and habitat provision (Gentry *et al.* 2020; Theuerkauf *et al.* 2019, 2022). In particular, oysters are one of the oldest farmed bivalves and they lead molluscan aquaculture production worldwide (Botta *et al.* 2020). Globally, the Pacific oyster (*Crassostrea gigas*) is the most important farmed species, with introductions in 66 countries for cultivation outside of its native range (Herbert *et al.* 2016; Ruesink *et al.* 2005). In some cases, notably in Europe, the establishment of *C. gigas* populations has led to their status as an important invasive species, with significant impacts on native biodiversity and ecosystem functioning (Ruesink *et al.* 2005). Using native oysters, where possible, has been suggested to be a better alternative to minimize ecological and environmental impacts, while supporting socio-economic development (Herbert *et al.* 2016).

The Eastern oyster, *Crassostrea virginica*, is one such native species which has been the target of considerable efforts throughout its range. In Atlantic Canada, production of the Eastern oyster has seen a strong expansion (>15% per year), with a value of ∼$31 million (CAD) in 2017 (an increase of 25% from 2016). Aquaculture production has also increased elsewhere *C. virginica*'s natural range, which extends from Northern New Brunswick in Canada, to the Gulf of Mexico in the United States with the adoption of floating aquaculture techniques. *C. virginica* is a particularly interesting species for aquaculture production due to its long shelf life and broad range of environmental tolerances (Marshall *et al.* 2021). Currently, nearly all Canadian production is based on wild seed collection and no domesticated strain is commercially available. As demand continues to rise, improved aquaculture approaches will be necessary to sustain oyster production.

Genetic improvement via selective breeding is one of the most important approaches currently used to enhance production of farmed species (Yáñez *et al.* 2022b). Selective breeding practices are widely used for genetic improvement of livestock but their adoption in aquaculture has been slower, especially for shellfish (Gjedrem *et al.* 2012; Houston *et al.* 2020). While industrialization of aquatic farming is relatively recent, it is expanding rapidly and the demonstrated responses to selection across farmed aquatic species (Gjedrem and Rye 2018) show that selective breeding can increase efficiency and profitability of production. Traditional selective breeding programs typically rely on the measurement of production traits (e.g. growth) and detailed pedigree information to predict the genetic merit of selection candidates for genetic improvement (Dufflocq *et al.* 2017). Genetic evaluations performed using pedigree-based best linear unbiased predictor (P-BLUP) are well suited for traits that can be directly recorded in the selection candidates. However, the P-BLUP approach presents some limitations in terms of prediction accuracy when the trait of interest is difficult or impossible to measure directly in the selection candidates (e.g. meat quality and disease resistance) and must be assessed via sib-testing. This may limit the rate of genetic progress reached on each generation.

Genomic selection (e.g. genomic-BLUP or G-BLUP) provides a valuable alternative by allowing the incorporation of genomic information into selective breeding programs of farmed species to accelerate genetic progress (Georges *et al.* 2019; Houston *et al.* 2020). Studies have demonstrated a considerable increase in the accuracy of genomic predictions compared with pedigree-based predictions for various traits, including those associated with growth and resistance to pathogens (Barría *et al.* 2018; Ødegård *et al.* 2014; Yoshida *et al*. 2018). Genomic selection is nowadays routinely applied for the genetic improvement of some aquatic farmed species, including salmon, trout, and tilapia (Houston *et al.* 2020; Lhorente *et al.* 2019; Verbyla *et al.* 2022; Yáñez *et al.* 2022a; Yáñez *et al.* 2020). For example, recent implementation of genomic selection in commercial Atlantic salmon (*Salmo salar* L.) farming demonstrated substantial increases in prediction accuracy when using genomic estimated breeding values (gEBVs) and accelerated rates of genetic gain for key production traits (e.g. disease resistance, harvest weight, color) with clear economic benefits (Verbyla *et al.* 2022). Moreover, selection based on genomic data can actually lower the rate of inbreeding while delivering higher genetic gains because of the improved resolution on the prediction of the Mendelian sampling term of gEBVs, increasing differentiation between siblings, and reducing their co-selection when compared to pedigree-based selection (Daetwyler *et al.* 2007; Sonesson *et al.* 2012). This is especially important in aquaculture species, for which selection intensities are typically high due to the prolific reproductive outputs of most marine aquaculture species (Houston *et al.* 2020).

The extensive application of genomic selection is often limited by the availability of affordable tools for rapid high-throughput genotyping such as species-specific SNP panels. Dense SNP panels have been developed and characterized for a number of high-value aquaculture species, predominantly finfish including Atlantic salmon (132K: Houston *et al.* 2014, 200K: Yáñez *et al.* 2016), rainbow trout (*Oncorhynchus mykiss*; 57K: Palti *et al.* 2015, 665K: Bernard *et al.* 2022), tilapia (*Oreochromis niloticus*; 58K: Joshi *et al.* 2018; 65K: Peñaloza *et al*. 2020; 50K: Yáñez *et al.* 2020, catfish (250K: Liu *et al.* 2014), pacu (*Piaractus mesopotamicus*) and tambaqui (*Colossoma macropomum*) (30K: Mastrochirico-Filho *et al.* 2021), and recently for the economically important Pacific white shrimp as well (*Litopenaeus vannamei*; 50K: Garcia *et al.* 2021). These resources have enabled accurate genomic prediction and genetic improvement for commercially-relevant traits in several aquaculture species (Bangera *et al.* 2017; Joshi *et al.* 2020; Ødegård *et al.* 2014; Vallejo *et al.* 2018; Yoshida *et al.* 2018, 2019; reviewed in Houston *et al.* 2020). For oysters, a high-density (200 K) SNP array for the Pacific oyster (*Crassostrea* gigas) has been released (Qi *et al.* 2017), as well as a medium-density combined-species array for the Pacific oyster (27 K) and the European flat oyster (11 K; *Ostrea edulis*) (Gutierrez *et al.* 2017) that has been used to test genomic selection for growth traits and disease resistance in the Pacific oyster (Gutierrez *et al.* 2018, 2020). For the Eastern oyster, genomic resources have been relatively limited. A validated panel of 58 SNPs was previously released, primarily for use in parentage analysis and studies of population structure (Thongda *et al.* 2018). More recently, high-density SNP arrays were published for *C. virginica* in the United States (Guo *et al.* 2023). The potential of genomic selection in oysters is well established; thus, the availability of genomic tools could have enormous positive impacts on the Eastern oyster aquaculture industry.

The objective of this study was to design and validate the first high-density (200 K) SNP array specific to the Eastern oyster (*C. virginica*) in Canada. This chip will enable rapid and cost-effective high-throughput genotyping for a broad range of applications, including genome-wide association studies, population genetic analyses, monitoring of genetic diversity in wild and farmed populations, and genomic selection to accelerate genetic progress. We also demonstrate the power of cost-effective low-coverage whole-genome sequencing (lcWGS), which has been shown to be an optimal approach for accurately inferring allele frequencies (Buerkle and Gompert 2013), for SNP discovery. The availability of a large SNP panel is especially important for oysters, since high recombination rates (and consequently weak linkage disequilibrium), which is characteristic of oyster genomes (Hollenbeck and Johnston 2018), may limit the detection of genotype–phenotype associations and inhibit accurate imputations from a smaller number of SNP markers. Furthermore, oyster genomes have a particularly high degree of polymorphism (Zhang *et al.* 2012) allowing for the detection of a large number of markers for panel development. With the availability of a high-quality *C. virginica* genome assembly (NCBI PRJNA376014, GCA_002022765.4, C_virginica-3.0), we were able to map SNPs onto the genome, allowing for the targeted selection of variants with an even distribution along the genome. The development of our high-density SNP chip will complement the resources developed for US populations by extending the availability of genomic tools to cover the northern limits of the species' distribution. This resource will provide a much-needed opportunity to accelerate Eastern oyster production in Canada by enabling genomic predictions, as well as encouraging the incorporation of genomic tools for monitoring breeding populations (e.g. inbreeding, relatedness) and facilitating further genomic research on wild and cultured populations.

## Materials and methods

### Sampling, DNA extraction, and sequencing

Samples were collected from crosses of a *C. virginica* strain that is currently in development at L’Étang Ruisseau Bar, Ltd. (ERB) in Shippagan, New Brunswick, Canada. This strain was founded from 464 oysters from 11 wild populations sampled in 2014 across New Brunswick and bred in a series of interpopulation crosses in winter 2015; a second generation was produced in 2018. The wild founder populations have been previously shown to exhibit high levels of within-population diversity and between-population differentiation (Bernatchez *et al.* 2019). A section of the adductor muscle was collected from 580 oysters from the F1 cohort and preserved in 95% ethanol during spawning in 2018. Genomic DNA was extracted from the muscle tissue using the NucleoMag Tissue Kit for DNA purification (Macherey-Nagel). DNA quality was checked on a 1% agarose gel electrophoresis and only samples that showed high molecular weight bands were retained for library preparation. Samples were then cleaned using Axygen magnetic beads with a ratio of 0.4:1 to retain only fragments >1 kb, following (Therkildsen and Palumbi 2017). DNA concentrations were measured using the QuantiT Picogreen dsDNA Assay Kit (Invitrogen) and all samples were normalized to 5 ng/μL. We then randomized and distributed all samples across 5 96-well plates and re-normalized samples to 1 ng/μL. Libraries were prepared for low-coverage whole-genome sequencing (lcWGS) using a protocol adapted from (Baym *et al.* 2015; Mérot *et al.* 2021; Therkildsen and Palumbi 2017). First, samples underwent a tagmentation reaction using enzymes from the Nextera kit in a 2.65 μL volume with ∼1 ng of DNA. We used a 2-step PCR protocol (8 + 4 cycles = 12 cycles in total) to add the Illumina adapter sequences with dual-index barcodes and amplify the libraries with the KAPA Library Amplification Kit and custom primers derived from the Nextera XT barcodes (using sets A- D; 384 dual-index combinations in total). Next, we used Axygen magnetic beads to purify the PCR products and perform size selection in 2 steps: (1) using a ratio of 0.5:1 and keeping the supernatant (for medium and short fragments), and (2) using a ratio of 0.75:1 and keeping the beads (for medium fragments). Final library concentrations were quantified using the QuantiT Picogreen dsDNA Assay Kit (Invitrogen) and the distribution of fragment sizes was analyzed using the Agilent BioAnalyzer for a subset of 10–15 samples per plate. We pooled equimolar amounts of 137–159 libraries for sequencing on 3 Illumina NovaSeq 6000 S4 lanes of paired-end 150 bp reads at the Centre d’Expertise et de Services Genome Québec (Montréal, QC Canada).

### SNP identification

We used the WGS sample preparation pipeline to clean and align sequence data (https://github.com/enormandeau/wgs_sample_preparation). Briefly, raw sequencing reads were trimmed and filtered for quality using fastp with default parameters (Chen *et al.* 2018). Trimmed sequences were then aligned to the Eastern oyster reference genome [https://www.ncbi.nlm.nih.gov/genome/398] using BWA-MEM with default parameters (Li and Durbin 2009) and filtered using samtools v1.8 [-q 10] (Li *et al.* 2009) to retain reads with a mapping quality >10. We used MarkDuplicates (default parameters; PicardTools v1.119) to remove duplicate reads and realigned around indels with GATK RealignerTargetCreator followed by IndelRealigner (default parameters; McKenna *et al.*, 2010). Overlapping read ends were then soft clipped using clipOverlap [–unmapped –storeOrig OC –stats] in bamUtil v1.1.14 (Breese and Liu 2013). The read with the highest quality score in overlapping regions was retained and any unmapped reads were removed using samtools v1.8 [-F 4] (Li *et al.* 2009). Genotype likelihoods were estimated from the aligned reads (in .*bam* format) using the GATK model (-GL 2) in ANGSD v0.923 (Korneliussen *et al.* 2014). Genotype calling was also performed with ANGSD, with posterior genotype probabilities estimated based on allele frequency as a prior (-doPost 1) and excluding genotypes with a posterior probability < 0.5 (-postCutoff 0.5). Sites that did not have data from at least 50% of individuals were excluded (-minInd 217). We retained biallelic SNPs with MAF > 0.01 (-minMAF 0.01), as well as sites with a minimum mapQ quality score of 30 (-minMapQ 30), a minimum Q-score of 20 (-minQ 20), and a total depth < 8,700 (-setMaxDepth 8700).

### SNP selection

**Fig. 1.**
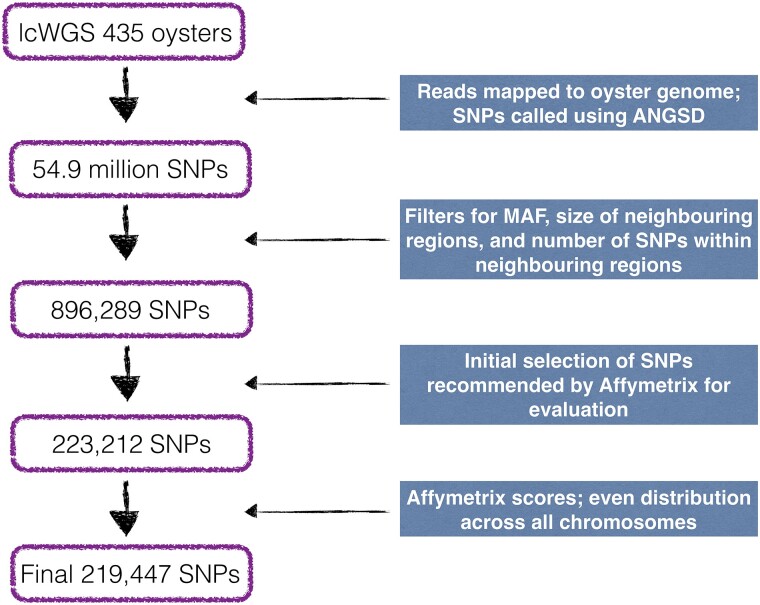
Summary of filtering steps from low-coverage whole-genome sequencing (lcWGS) of 435 oysters to selection of 219,447 polymorphic sites for the SNP array.

We filtered SNPs according to all possible combinations of the following criteria: (1) minimum MAF (0.05 and 0.1); (2) size of neighboring regions around each SNP of interest (20, 25, or 30 bp); and (3) maximum number of SNPs permitted in the neighboring regions (0, 1, or 2). The best SNPs for the purpose of the Affymetrix array (i.e. the most stringent filtering of all 3 criteria) would have a MAF ≥ 0.1 and 0 SNPs in the neighboring 30 bp on each side of the SNP of interest. With this approach, multiple subsets of SNPs were generated with different degrees of filtering stringency; SNPs that passed the most stringent combination of criteria were present in all subsets. For a given SNP, we counted the number of subsets to which it belonged and ranked all SNPs by the number of sets in which they were present (i.e. SNPs that were present in more sets passed more stringent criteria and were therefore assigned a higher ranking). SNPs were given a priority number starting from 1 for the highest priority and increasing as priority got lower, as is required by Affymetrix for SNP evaluation. This list and associated information, including chromosome name and position, the flanking sequences, and central SNP alleles, and the priority number was sent to Affymetrix for evaluation. Based on the scores received and our own priority numbers, we selected the best SNPs for printing on the Affymetrix Axiom® myDesign Custom Array including ∼200 K SNPs, while also ensuring that the distribution of SNPs retained was even across all chromosomes after correcting for chromosome length (see Fig. 1 for a summary of the filtering steps and selection criteria). SNPs with MAF values greater than 0.1 were prioritized, but in some cases, SNPs with MAF below 0.1 were chosen to avoid retaining SNPs with more neighboring SNPs or smaller SNP-free neighboring regions.

### SNP validation

The SNPs printed on the array were tested and validated in ∼4,500 oysters across 2 generations from the ERB strain. We genotyped all F1 oysters, which represent the progeny of crosses between 11 wild populations (39 crosses), including the 435 oysters that were sequenced for SNP discovery. The F1 broodstock oysters were subsequently bred in 82 crosses. A total of 3,000 F2 oysters were sampled randomly in November 2020 and muscle tissues were preserved in 95% ethanol. In addition, ∼1,000 F2 oysters were selected as broodstock based on measured traits (e.g. shell length, shell width, and weight). Muscle tissue samples were taken from these breeders during spawning in January 2021. DNA extractions were performed on all 4,000 F2 oysters as described above (see “DNA Extraction and Sequencing”) and genotyped on the SNP chip. Genotyping was carried out at the Génome Québec Centre d'expertise et de services (Montréal, QC, Canada) following standard protocols for the Axiom Affymetrix platform. Quality control (QC) analysis and genotype calling were performed using the Axiom® Analysis Suite Software (ThermoFisher, Affymetrix).

We computed minor allele frequencies (MAF) and observed and expected heterozygosity (H_o_ and H_e_, respectively), across the 2 cohorts using VCFTools (Danecek *et al.* 2011) and Plink v.1.90 (Purcell *et al.* 2007). The inbreeding coefficient (F_IS_) was estimated across loci as (H_e_—H_o_)/H_e_ and significant departures from Hardy–Weinberg equilibrium (HWE) were evaluated using Plink, with a Bonferroni adjustment for multiple comparisons (α = 0.05). LD decay was computed for each cohort independently up to a maximum distance of 500 kb between SNPs using PopLDdecay v.3.41 (Zhang *et al*. 2019).

### Parental assignment and Mendelian inheritance errors

Kinship was reconstructed using the R package “*Sequoia*” (Huisman 2017) with 545 SNPs in HWE and with MAF > 0.3 and filtered by LD. While a larger number of markers would increase the accuracy of kinship assignment to some extent, other studies have demonstrated that kinship can be estimated with high accuracy using a similar number of SNPs (Anderson and Garza 2006; Dussault and Boulding 2018; Premachandra *et al.* 2019). *Sequoia* uses the year of birth information of each individual to discriminate between generations accordingly. In addition, to minimize the risk of assignment error, pedigree reconstruction was performed in 62 different groups according to the outcross records of each F1 animal and the traceability of the outcross from which each F2 animal originated. Each group contained 6 to 8 potential parents, ranging from 1 to 5 females and 3 to 6 males per group. After kinship reconstruction, Mendelian inheritance errors (MIE) per SNP were estimated using Plink v.1.90 (Purcell *et al.* 2007) for all SNPs that passed quality control. MIEs are errors detected when the offspring have genotypes or alleles that are inconsistent with the parental genotypes. Potential sources of MIEs include de novo mutations, presence of structural variants, genotyping errors, allele dropout, or false negatives in low-quality samples. Herein, we report individual-level MIE rates as the number of SNP errors per individual divided by the total number of called SNPs, and SNP-level MIE rates as the number of errors per SNP divided by the total number of individuals. Finally, we identified SNPs that may be considered as having an abnormally high MIE rate as SNPs with an error rate that exceeded an upper threshold defined by the third quartile range (QR3) plus 1.5 * the interquartile range (IQR).

## Results

### SNP identification and selection

Low-coverage whole-genome sequencing (lcWGS) of 435 oysters yielded an average of 19.65 million reads per sample. This represented an average of 2.64 Gbp per sample, which translates to an average coverage of 3.86 × (sd 1.33X). Following all data cleaning and preparation steps with the WGS sample preparation pipeline (trimming, de-duplicating, re-aligning indels, and removing paired-end overlaps), 85.2% of the raw reads were mapped to single positions on the genome and were used for calling SNPs. After applying filters and calling genotypes in ANGSD, we retrieved a total of 54,945,771 SNPs.

**Fig. 2.**
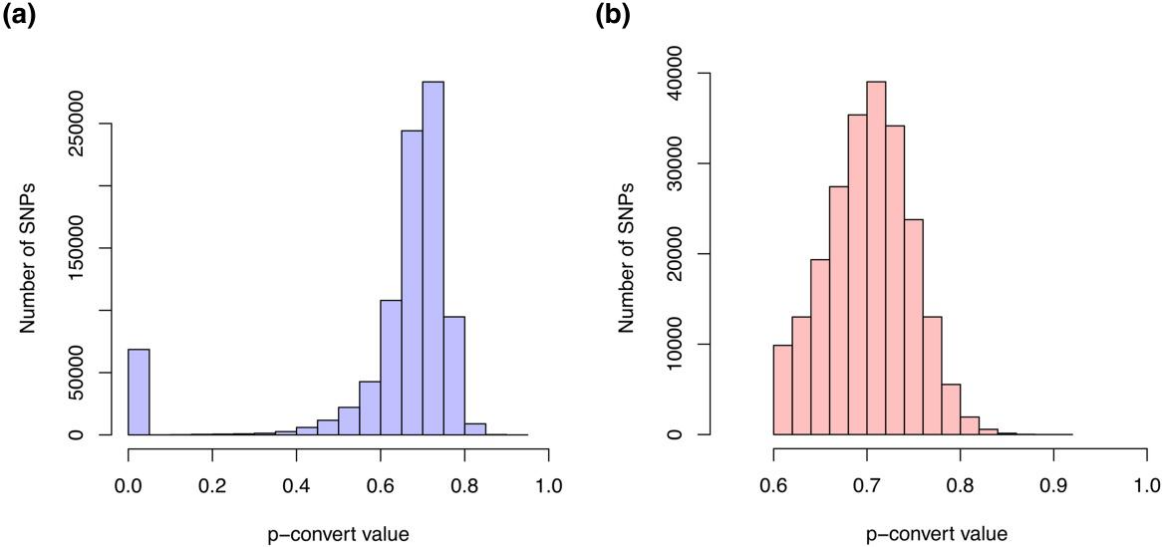
Distribution of *p*-convert values for a) all candidate probes (*n* = 896,289) and b) probes that were recommended and evaluated for the SNP array (*n* = 223,212).

We then filtered this full list of SNPs according to combinations of (1) minimum MAF, (2) size of neighboring region around each SNP of interest, and (3) maximum number of SNPs allowed in each neighboring region, which generated 18 sets of SNPs with more or less stringent parameters. A total of 159,800 SNPs passed the most stringent combination of criteria (MAF > 0.1 and 0 SNPs in neighboring regions of 30 bp on each side of the SNP of interest). Across all 18 sets, a list of 896,289 SNPs was retained, with an average rate of missing SNP genotypes of 20% across the entire data set. These SNPs were ranked by priority and sent to Affymetrix for evaluation. Of these candidate SNPs, a total of 793,104 were categorized by Affymetrix as “recommended” or “neutral”, 91% of which had a *p*-convert value > 0.6 (Fig. 2a). From this list, a subset of 223,212 of the SNPs that were recommended by Affymetrix were evaluated for inclusion on the chip, 100% of which had *p*-convert values > 0.6 (Fig. 2b). Based on the scores and our priority list, a total of 219,447 SNPs were retained and used for the creation of the chip (Fig. 1, Supplementary Table 1). All of these SNPs had a MAF > 0.05 and 198,786 SNPs (91%) had a MAF > 0.1.

### SNP validation

**Fig. 3.**
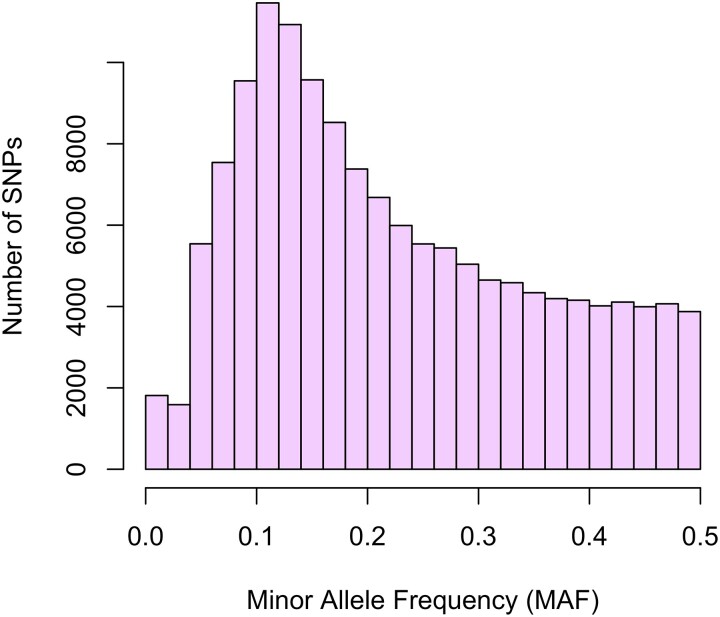
Minor allele frequency (MAF) distribution for all samples that passed quality control filtering (*n* = 4,350).

**Fig. 4.**
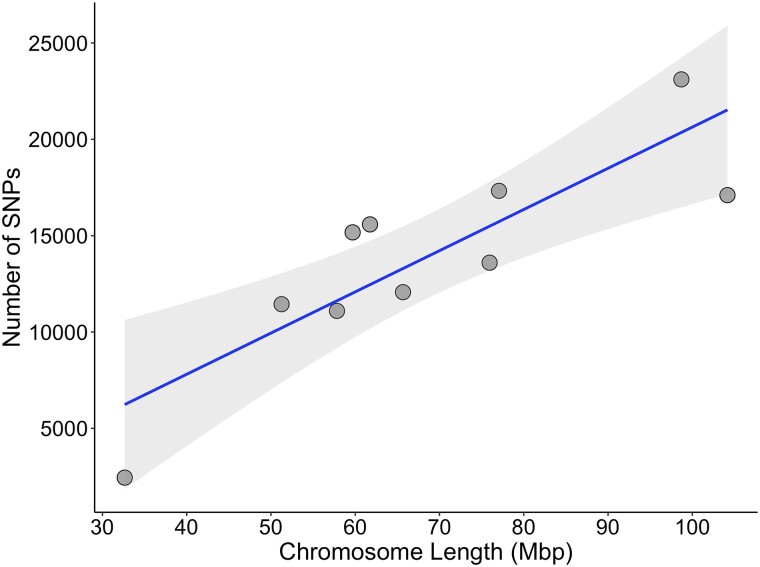
Relationship between the number of SNPs and chromosome length. The correlation coefficient between the number of SNPs and chromosome length is *r* = 0.8 (*P* = 0.006).

**Fig. 5.**
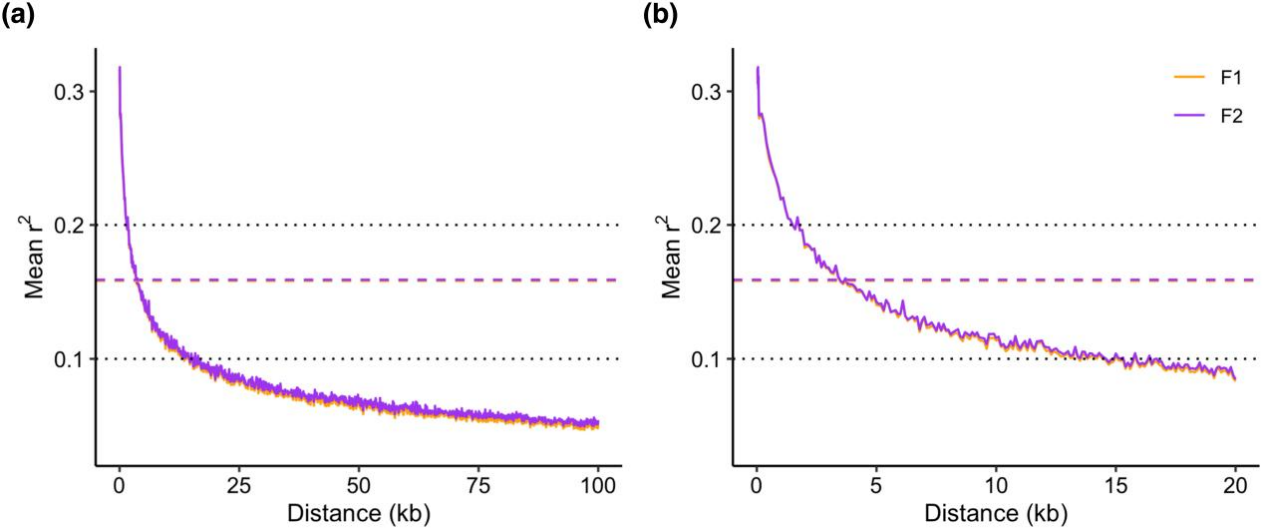
Plot of LD decay for F1 and F2 cohorts up to a maximum distance of of a) 100 kb and b) 20 kb. The dotted lines denote pairwise *r*^2^ values of 0.2 and 0.1, and the dashed lines indicate the *r*^2^ value that is half the maximum *r*^2^ value for both F1 and F2 cohorts.

We genotyped 4,356 oysters on the chip, including 558 from the F1 cohort and 3,798 from the F2 cohort. Six oysters were excluded after filtering for a minimum individual genotyping call rate of 90%. The remaining 4,350 oysters all had a dish QC (DQC) value > 0.856. A total of 144,570 SNPs had a minimum call rate of 90%, with a median minor allele frequency (MAF) of 0.2 (Fig. 3). Out of the 144 K successfully genotyped SNPs, the majority (96%) were polymorphic, with MAF > 0.05 for both sampled generations (Table 1). The overall conversion rate on the chip was 63%, which is slightly lower than the mean *p*-convert value of 0.7; transition SNPs (A/G and C/T) comprised about 58% of the successfully converted SNPs (Table 2). The correlation between the number of polymorphic SNPs (MAF > 0.05) within each chromosome and the total chromosome length was strong (*r* = 0.8, *p* = 0.006; Fig. 4) indicating that the validated SNPs are evenly distributed across the chromosomes on the eastern oyster genome. Genetic diversity in F1 oysters was replicated in the F2 progeny, with similar expected heterozygosity (H_e_) across both groups (Table 1). Moreover, observed and expected heterozygosity proportions and per-locus F_IS_ estimates were similar in both cohorts, with an average F_IS_ across all polymorphic loci close to 0 (∼0.02–0.03; Table 1). A total of 130,148 SNPs (93%) and 107,614 SNPs (78%) were in HWE in the F1 and F2 groups, respectively (Table 1). Mean LD between marker pairs was similar between the F1 and F2 cohorts and was low overall (Fig. 5). Maximum mean LD (*r*^2^) at the shortest inter-SNP distance (30 bp) was ∼0.32 for both groups and showed a moderate to low decay of LD with increasing distance between SNP pairs (Fig. 5, Table 3). For both F1 and F2 groups, pairwise *r*^2^ declined to half the maximum value (∼0.16) and to < 0.1 at a distance of approximately 3.5 and 15 kb, respectively (Fig. 5).

**Table 1.** Descriptive statistics from 2 cohorts (F1 and F2) that were genotyped on the SNP chip: *n*, total number of individuals genotyped; nQC, number of individual samples that passed quality checks (90% of SNPs genotyped); HWE, number (N) and percent (%) of SNPs that were in Hardy–Weinberg equilibrium; MAF, number (N) and percent (%) of SNPs with minor allele frequency greater than 0.05, 0.1, and 0.2; *H*_o_, observed heterozygosity; *H*_e_, expected heterozygosity; *F*_IS_, per-locus inbreeding coefficient.

	*n*	nQC	Call rate	MAF > 0.05	MAF > 0.1	MAF > 0.2	HWE*	H_o_**	H_e_**	F_IS_**	Min *F_IS_*	Max *F_IS_*
				*N* (%)	*N* (%)	*N* (%)	*N* (%)					
F1	558	553	144,570	138,992 (96.14)	117,853 (81.52)	69,745 (48.24)	130,148 (93.64)	0.31 ± 0.13	0.32 ± 0.12	0.029 ± 0.12	−0.89	1
F2	3,798	3,797	144,570	138,820 (96.02)	118,487 (81.96)	70,852 (49.01)	107,614 (77.52)	0.32 ± 0.13	0.33 ± 0.12	−0.023 ± 0.12	−0.85	1

*% of SNPs segregating in HWE out of the number of polymorphic SNPs (MAF > 0.05).

**Ho, He, and F_IS_ estimated on SNPs with MAF > 0.05 in each generation (∼139 K).

**Table 2.** Counts of SNP types for all polymorphic SNPs (MAF > 0.05).

SNP type	On-chip		Converted (MAF > 0.05)		Conversion rate
	Count	%	Count	%	
Transitions					
A/G	61,504	28	40,499	29	0.66
C/T	61,594	28	40,626	29	0.66
Transversions					
G/T	23,892	11	14,368	10	0.60
A/C	24,310	11	14,754	11	0.61
A/T	35,852	16	20,211	15	0.56
C/G	12,295	6	8,478	6	0.69
Total	219,447	100	138,936	100	0.63

**Table 3.** Average linkage disequilibrium (*r*^2^) between SNPs at increasing pairwise distances.

Generation	Distance (bp)	Mean *r*^2^
F1	100	0.280
F2	100	0.282
F1	1,000	0.219
F2	1,000	0.219
F1	5,000	0.140
F2	5,000	0.143
F1	10,000	0.114
F2	10,000	0.116
F1	15,000	0.102
F2	15,000	0.104

### Parental assignment and Mendelian inheritance errors (MIE)

**Fig. 6.**
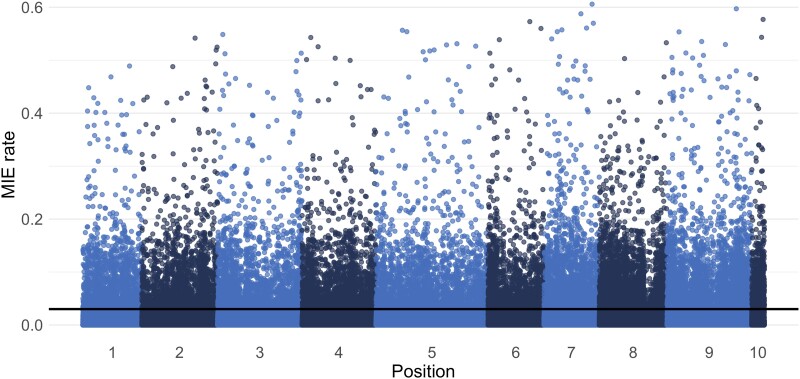
Manhattan plot of MIE rates per SNP along the *C. virginica* genome with the threshold determined by Q3 + 1.5*IQR indicated by the black line.

**Fig. 7.**
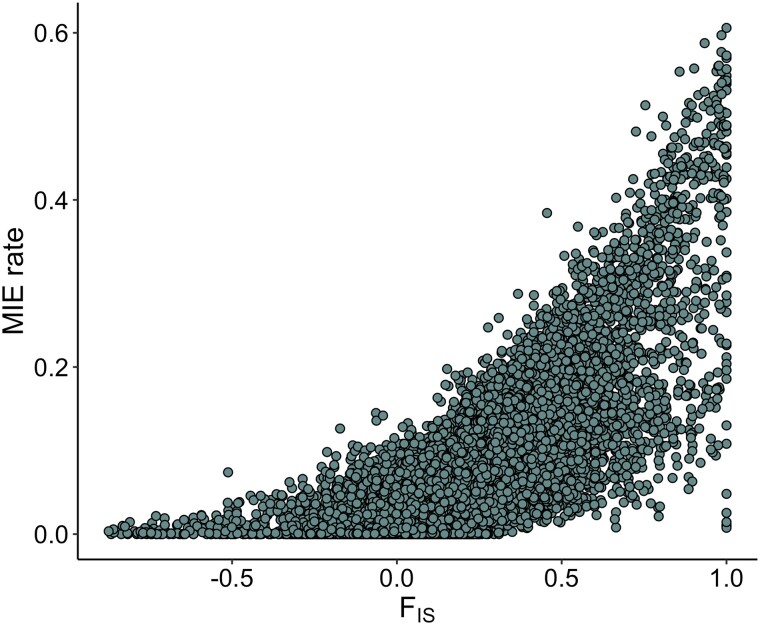
Mendelian inheritance error (MIE) rates plotted as a function of the per-locus inbreeding coefficient (F_IS_) for all called SNPs (Pearson correlation *r* = 0.76).

A total of 2,636 F2 animals (68%), corresponding to a total of 230 full-sib families, 115 maternal half-sib families, and 161 paternal half-sib families, were reconstructed for full parentage (sire and dam). The resulting pedigree was used to estimate Mendelian inheritance errors (MIEs) across individual oysters and across all genotyped SNPs that passed quality control (∼144 K). At the individual level, the average MIE rate was 0.015 (+- 0.002), with a maximum error count for an individual of 6,270 (out of 144,570 called SNPs; individual MIE rate = 4.3%). MIEs were distributed across each of the 10 chromosomes (Fig. 6). The median rate of genome-wide MIEs per SNP was 0.0023, and 103,918 SNPs (72% of called SNPs) had a MIE rate of < 0.01 (Table 4). Using the third quartile of MIE rates + 1.5* the interquartile range as a cut-off for excluding SNPs, which corresponds to a maximum error rate of 0.03, a total of 125,627 SNPs (87% of called SNPs) were retained (Table 4, Fig. 6). To investigate the cause of extreme MIE rates (i.e. up to a maximum of 0.6 for a given SNP), we plotted MIE rates per SNP against the inbreeding coefficient (F_IS_), which revealed an increase in MIE rate with increasingly positive F_IS_ values (Pearson correlation *r* = 0.76; Fig. 7). For the 103,918 SNPs with an error rate < 0.01, there was no correlation between MIE rate and F_IS_ (*r* = 0.10), whereas for loci with an error rate > 0.01, there was a strong correlation (*r* = 0.78) (Supplementary Fig. 1). This heterozygote deficiency for SNPs exhibiting high rates of MIEs may suggest the presence of null alleles; indeed, examination of the specific inheritance error types provided by Plink indicated a much higher proportion of MIE errors that are consistent with null alleles (Table 5).

**Table 4.** Number and percentage of all called SNPs (144 K) retained following MIE cut-offs.

MIE rate cut-off	Number (%) of SNPS retained
0	36,794 (25%)
0.01	103,918 (72%)
0.02	118,697 (82%)
0.03*^a^*	125,627 (87%)
0.05	132,251 (91%)

Cut-off based on the third quartile + 1.5 * the inter-quartile range.

**Table 5.** Proportion of MIEs of each type according to Plink error codes. Bold data indicate errors that could be caused by null alleles.

MIE code	Description of MIE	Proportion of errors
1	A/A × A/A → A/B	0.0084
2	B/B × B/B → A/B	0.094
**3**	B/B × */* → A/A	**0.22**
**4**	*/*×B/B → A/A	**0.22**
5	B/B × B/B → A/A	0.0090
**6**	A/A × */* → B/B	**0.23**
**7**	*/*×A/A → B/B	**0.22**
8	A/A × A/A → B/B	0.0050

## Discussion

The 200 K SNP array designed and validated in this study represents the first SNP panel characterized for *C. virginica* in Canada to date and will be an essential asset for the oyster aquaculture industry. The recent assembly of a high-quality chromosome level reference genome for the Eastern oyster [https://www.ncbi.nlm.nih.gov/genome/398] enabled us to achieve an even distribution of SNPs across all ten Eastern oyster chromosomes, ensuring genome-wide coverage of genetic variation represented on the chip. After genotyping more than 4,000 individual oysters spanning 2 generations, the majority of the successfully called SNPs (144 K) were polymorphic (96%), yielding a total of 139 K high-quality SNPs that were successfully genotyped on the chip (out of 219 K, ∼63% conversion rate). A similar conversion rate was observed on the high-density SNP panel of similar size developed for the Pacific oyster (*C. gigas*), in which 133 K SNPs out of 190 K (70%) were successfully genotyped (Qi *et al.* 2017). The high degree of polymorphism and successful conversion rate also demonstrates the merit of the low-cost low-coverage whole-genome sequencing approach used to obtain accurate allele frequencies for optimal selection of SNP markers for evaluation. The availability of this SNP array specific to *C. virginica* will augment the currently limited genomics toolbox for this species and will be advantageous for commercial applications in Canada where production has been expanding rapidly, including genome-wide association studies (GWAS) and genomic selection. Additional sampling and genotyping of oysters from populations outside of the geographic area sampled for this study will be needed to evaluate marker conversion rate and commercial applicability in populations that were not used for SNP array design.

Although MIEs were observed across the *C. virginica* genome, a large proportion of the 144 K SNPs with called genotypes exhibited low error rates (median MIE rate = 0.002), with 72% of these SNPs having an error rate lower than 1%, suggesting that the majority of called SNPs segregate under Mendelian inheritance. However, a nontrivial number of SNPs exhibited high error rates, up to a maximum MIE rate of 60%. While we acknowledge that the MIEs detected in this study are contingent on the results of the performed kinship reconstruction and assumes that parents are correctly genotyped, the protocol we used minimizes the risks of generating wrong assignments (i.e. using a priori information from tracked crosses, selecting markers with higher MAF and low linkage), and complies with the recommendations in both the *Sequoia* manual and literature (Dussault and Boulding 2018; Huisman 2017; Premachandra *et al.* 2019). The association between high MIE rates and heterozygote deficiency suggests that the errors observed are not due to random genotyping errors, but rather may be driven by the presence of null alleles. Null alleles result in an excess of homozygotes and have been associated with apparent non-Mendelian segregation observed at microsatellite loci developed for *C. virginica* (Reece *et al*. 2004) and were implicated as a major source of MIEs in a SNP panel designed for the same species (Guo *et al*. 2023). Furthermore, copy number variation has been suggested as a pervasive feature in the *C. virginica* genome (Modak *et al*. 2021) and can generate MIEs (Arias *et al*. 2022). Therefore, SNP loci with elevated MIEs are candidates for further exploration for the presence of null alleles or copy number variants. Heterozygote deficiency has long been an observed feature of genetic studies in oysters (Hare *et al*. 1996, Hedgecock *et al*. 1996), and this study revealed an explicit link between heterozygote deficiency (as measured by F_IS_) and deviations from Mendelian segregation, which cannot be induced by selective forces.

The extent of LD observed in F1 and F2 cohorts was low overall and mean pairwise *r*^2^ declined at a moderate rate with increasing physical distance. Relatively low levels of LD are expected for *C. virginica* given the high recombination rates characteristic of oyster genomes (Hollenbeck and Johnston 2018) and our estimates are consistent with levels of LD and general patterns of LD decay observed in *C. gigas* populations (Gutierrez *et al.* 2017; Hu *et al.* 2022; Zhong *et al.* 2017). LD is important in certain applications such as GWAS, in which strong linkage between SNPs can facilitate the detection of significant associations with traits, even if causal variants are not genotyped (i.e. indirect associations; Bush and Moore 2012) and accurate genomic predictions can be made with fewer SNPs when LD is high. The moderate persistence of LD and the high density of markers that we targeted for our SNP array, with an average of 150 SNPs for every 1 Mbp across all 10 chromosomes, are promising for future selective breeding efforts as the resolution should be sufficient to successfully detect genotype-phenotype associations and obtain accurate genomic predictions.

The parents of the F1 oysters that were used to design the SNP array originated from 11 bays in New Brunswick, Canada, for which the population genetic structure has been studied (Bernatchez *et al.* 2019). This previous work identified strong genetic differentiation among populations, including 6 major genetic clusters in this region, and genetic associations with environmental conditions. This strategy of SNP discovery generated a panel of markers that is representative of the genetic variation present across multiple locations and thus should be applicable to Eastern oysters originating from diverse populations in Canada. Given that we focused on populations at the northern range limit of *C. virginica*, our SNP chip complements that which was recently published for US populations (Guo *et al.* 2023). Indeed, the genetic composition between Canadian populations and populations further south may differ substantially, as a north-south genetic break has been observed on the Eastern Scotian Shelf for other marine species distributed along the Northwestern Atlantic coast (Stanley *et al.* 2018, Lehnert *et al*. 2019, Dorant *et al.* 2022). By extending the coverage of high-density SNP arrays into the most northern part of the Eastern oyster distribution with our SNP chip, genomic resources are now available throughout the entire species' range.

## Conclusions

This manuscript presents the first high-density (∼200 K) SNP array designed specifically for the Eastern oyster (*Crassostrea virginica*) in Canada, a species for which aquaculture production is increasing rapidly and genomic tools to sustain this growth are needed. The low-coverage whole-genome sequencing approach used here offered a low-cost and effective method for the discovery of a large number of highly polymorphic SNPs for panel development. Integration of the *C. virginica* reference genome allowed for the selection of SNPs that were evenly spaced along the entire genome. Indeed, validation of the SNP chip by genotyping >4,000 oysters across 2 generations of crosses showed that the 144 K successfully converted SNPs were evenly distributed across all 10 chromosomes. The relative persistence of LD with increasing physical distance between markers and the high density of SNPs targeted suggest that this panel is suitable for achieving high prediction accuracy. Analysis of MIEs revealed a large proportion of SNPs on the chip that segregate under Mendelian inheritance. The detection of elevated rates of unexpected genotypes at some SNP loci may be attributed to heterozygote deficiencies resulting from null alleles and highlight candidates for further investigation of copy-number variation.

The availability of this SNP array extends the coverage of genomic resources for *C. virginica* into the most northern limits of its distribution and will facilitate further genomics research, including the application of GWAS to identify variants associated with economically and ecologically important traits. In particular, this chip will be of great importance for the future of oyster aquaculture in Canada, as it will allow the evaluation of potential quantitative trait loci (QTL) and to unravel the genomic architecture of economically important traits, evaluate genomic breeding values, establish genomic prediction protocols, assess the adequate number of markers required to optimize resources, perform population genomic assessments, and conduct low-cost genomic selection for accelerated genetic gains.

## Supplementary Material

jkad071_Supplementary_Data

## Data Availability

Genome position and probes of SNPs included in the SNP array have been submitted to the figshare online repository as Supplementary Table 1: https://doi.org/10.25387/g3.21422661. DNA sequence reads for the F1 oysters used to design the chip (*n* = 435) were submitted to the National Center for Biotechnology Information (NCBI) Sequence Read Archive (SRA) under BioProject PRJNA933941. Supplemental material available at G3 online.
