# Supplementary material for: Design and validation of a high-density single nucleotide polymorphism array for the Eastern oyster (Crassostrea virginica):
Source: G3 (Bethesda). 2023 Mar 26;13(6):jkad071. doi: 10.1093/g3journal/jkad071 (PMC10234413; doi:10.1093/g3journal/jkad071)
Supplement: jkad071_Supplementary_Data [file jkad071_supplementary_data.zip › Figure_S1_G3-2023-404127.docx]

(a)


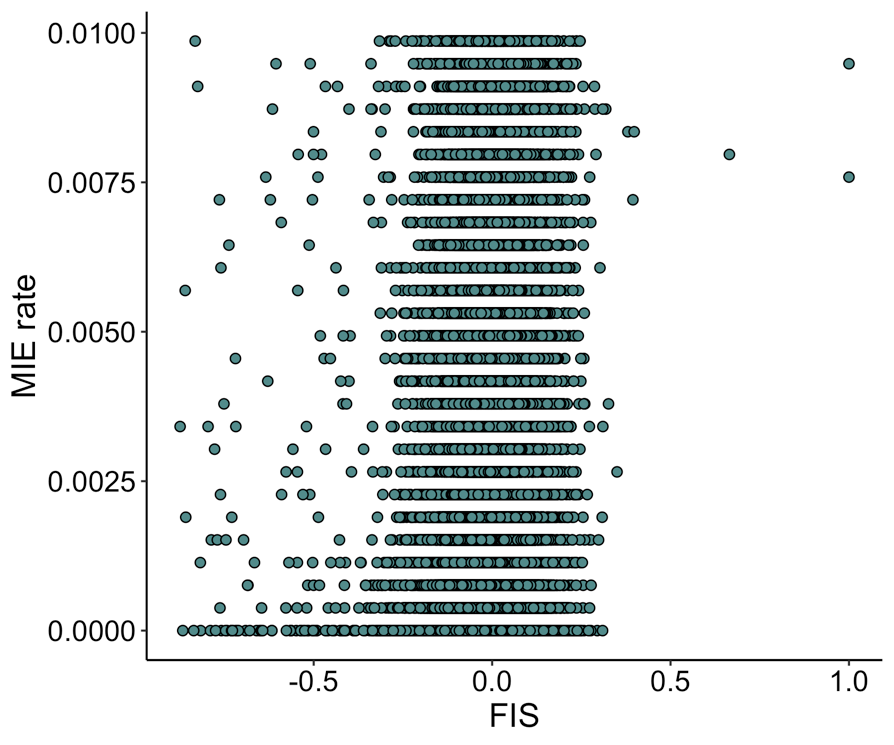


(b)


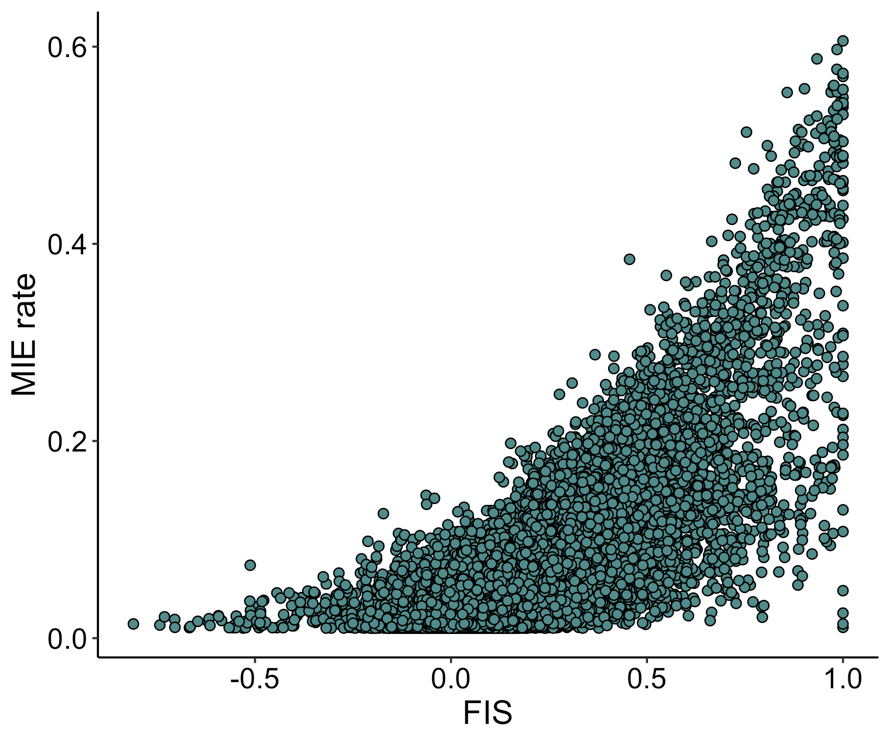


**Figure S1.** Mendelian inheritance error (MIE) rates plotted as a function of the inbreeding coefficient (F_IS_) for (a) SNPs with a MIE rate <= 0.01 (Pearson correlation *r* = 0.10), and (b) SNPs with a MIE rate > 0.01 (Pearson correlation *r* = 0.78).
